# FlexiMIND: Combined Pretrained Vision Features and Local Structure for Training-Free Deformable Medical Image Registration

**Published:** 2026-10-25

**Authors:** Mojtaba Safari, Mingzhe Hu, Shansong Wang, Suyang Xi, Joshua Lorenz, Sean Pitroda, Aytekin Oto, Ralph R. Weichselbaum, Xiaofeng Yang

## Abstract

Objective. Deformable image registration remains challenging because corresponding anatomy may exhibit different intensity relationships. Hand-crafted modality-robust descriptors such as Modality-Independent Neighborhood Descriptor with Self-Similarity Context (MIND-SSC) and features from pretrained vision encoders provide distinct representations for correspondence estimation, but how they should be combined remains unclear. We investigate whether tighter integration improves registration over a sequential, scale-separated design. Approach. We introduce FlexiMIND, a training-free and label-free two-stage framework in which frozen FlexiCT-2D encoder features establish contextual correspondence and MIND-SSC refines the deformation within ConvexAdam. Five alternative feature-coupling strategies were evaluated under a protocol across CT-CT (DIR-Lab 4DCT 4DCT, 10 cases), MR-MR (ACDC, 50 patients), and MR-CT (Learn2Reg Abdomen MR-CT, 8 pairs) registration. Performance was assessed using target registration error (TRE), Dice similarity coefficient (Dice), SDlogJ, and local folding. Main results. FlexiMIND achieved a TRE of 1.27 mm on DIR-Lab 4DCT, a DSC of 0.807 on ACDC, and an organ-averaged DSC of 0.825 on Abdomen MR-CT. Across 15 coupling-strategy dataset comparisons, only four outperformed the reference method, with a maximum absolute improvement of 0.0043 in Dice, whereas degradation reached 20.6 % in TRE and 37.9 % in Dice. Changes in registration accuracy were closely associated with changes in deformation-field regularity, and strategies that better preserved regularity generally remained closest to the reference performance. Landmark-level analysis showed that the pretrained encoder stage primarily reduced large correspondence errors rather than uniformly improving local precision. Code is available upon acceptance at https://github.com/mosaf/FlexiMIND.

